# Genomic Analysis and Secondary Metabolites Production of the Endophytic *Bacillus velezensis* Bvel1: A Biocontrol Agent against *Botrytis cinerea* Causing Bunch Rot in Post-Harvest Table Grapes

**DOI:** 10.3390/plants10081716

**Published:** 2021-08-20

**Authors:** Kallimachos Nifakos, Polina C. Tsalgatidou, Eirini-Evangelia Thomloudi, Aggeliki Skagia, Dimitrios Kotopoulis, Eirini Baira, Costas Delis, Konstantinos Papadimitriou, Emilia Markellou, Anastasia Venieraki, Panagiotis Katinakis

**Affiliations:** 1 Laboratory of General and Agricultural Microbiology, Crop Science Department, Agricultural University of Athens, Iera Odos 75, 11855 Athens, Greece; k.nifakos@go.uop.gr (K.N.); polinatsal@gmail.com (P.C.T.); e.e.thomloudi@gmail.com (E.-E.T.); Angeliki.Skagia@warwick.ac.uk (A.S.); drkotopoulis@gmail.com (D.K.); 2 Department of Agriculture, University of the Peloponnese, 24100 Kalamata, Greece; delis@us.uop.gr; 3 Laboratory of Toxicological Control of Pesticides, Scientific Directorate of Pesticides’ Control and Phytopharmacy, Benaki Phytopathological Institute (BPI), 8 St. Delta Street, Kifissia, 14561 Athens, Greece; e.baira@bpi.gr; 4 Department of Food Science and Technology, University of the Peloponnese, 24100 Kalamata, Greece; kostas.papadimitriou@gmail.com; 5 Scientific Directorate of Phytopathology, Benaki Phytopathological Institute (BPI), 14561 Athens, Greece; e.markellou@bpi.gr; 6 Laboratory of Plant Pathology, Crop Science Department, Agricultural University of Athens, Iera Odos 75, 11855 Athens, Greece

**Keywords:** endophytic bacteria, metabolites, iturin A, surfactin, genome sequence, colonization, biological control, induced systemic resistance

## Abstract

Botrytis bunch rot caused by *Botrytis cinerea* is one of the most economically significant post-harvest diseases of grapes. In the present study, we showed that the bacterial strain Bvel1 is phylogenetically affiliated to *Bacillus velezensis* species. The strain Bvel1 and its secreted metabolites exerted an antifungal activity, under in vitro conditions, against *B. cinerea*. UHPLC–HRMS chemical analysis revealed that iturin A2, surfactin-C13 and -C15, oxydifficidin, bacillibactin, L-dihydroanticapsin, and azelaic acid were among the metabolites secreted by Bvel1. Treatment of wounded grape berries with *Bacillus* sp. Bvel1 cell culture was effective for controlling grey mold ingress and expansion in vivo. The effectiveness of this biological control agent was a function of the cell culture concentration of the antagonist applied, while preventive treatment proved to be more effective compared to curative. The strain Bvel1 exhibited an adequate colonization efficiency in wounded grapes. The whole-genome phylogeny, combined with ANI and dDDH analyses, provided compelling evidence that the strain Bvel1 should be taxonomically classified as *Bacillus velezensis*. Genome mining approaches showed that the strain Bvel1 harbors 13 antimicrobial biosynthetic gene clusters, including iturin A, fengycin, surfactin, bacilysin, difficidin, bacillaene, and bacillibactin. The results provide new insights into the understanding of the endophytic *Bacillus velezensis* Bvel1 biocontrol mechanism against post-harvest fungal pathogens, including bunch rot disease in grape berries.

## 1. Introduction 

Botrytis bunch rot (grey mold) caused by the necrotrophic fungal pathogen *Botrytis cinerea* is a major disease of grapevines. Infection of ripening berries leads to large losses in the quantity and quality of both table and wine grapes, either in the field or under post-harvest storage conditions [1].

Although grey mold control has been mainly achieved using synthetic fungicides, public concerns regarding their adverse effects on the environment and human health have prompted the search for alternative disease control methodologies [1]. Among several scientific approaches, a promising alternative for grey mold management is the use of microorganisms (e.g., *Trichoderma* spp., *Bacillus* spp., *Candida* spp., and *Aureobasidium pullulans*) as biological control agents (BCAs) [2,3,4].

In recent years, bacterial strains of the genus *Bacillus* have received great attention as biocontrol agents against post-harvest fungal pathogens that infect fruits and vegetables [5]. Various mechanisms have been implicated in the *Bacillus* spp. protective effect, which include competition for space and nutrients, antibiosis through the secretion of compounds exhibiting antimicrobial activities, secretion of enzymes that degrade the plant pathogen cell wall, and induction of host resistance [6].

Numerous studies on the plant protection mechanisms of microbial biocontrol agents have demonstrated that the biological control effect of *Bacillus* sp. species is associated with their capacity to produce a plethora of bioactive molecules, with the lipopeptides (LPs) being the most well studied [5]. *Bacillus* sp. biosynthesized lipopeptides can be generally classified into various families based on the peptide amino acid composition and the fatty acid chain length; with fengycins, surfactins, and iturins being the three best known families [7]. *Bacillus* sp. mutants defective in lipopeptide biosynthesis, as well as cell-free culture supernatant (CFCS) from these mutants’ strains, lost their activity to suppress post-harvest pathogenic fungi, suggesting that the secreted lipopeptides are involved in the biocontrol activity of these antagonistic *Bacillus* strains [8,9,10,11]. Furthermore, it has been demonstrated that lipopeptides such as iturin, bacillomycin, and fengycin, besides their strong and broad-spectrum antifungal and/or antibacterial activity [12], may also serve as elicitors of plant-induced systemic resistance [13,14,15,16,17].

Recent studies, have demonstrated that endophytic *Bacillus* sp. may be considered as effective BCA candidates counteracting post-harvest disease in fruit and vegetables [18]. Endophytic antagonistic *Bacillus* species have the ability to colonize plant interior tissues without causing any visible symptoms of disease to their plant hosts, while in most cases they contribute to the promotion of plant growth and protection against both biotic and abiotic stress factors [6]. Endophytic *Bacillus* may also be more likely to succeed as biocontrol agents as a result of their environmental adaptation to host plant metabolites and their ability to occupy ecological niches similar to fungal pathogens, such as *B. cinerea,* that exhibit a latent or necrotrophic behavior [18]. Recent studies exploring the biocontrol capacity of endophytic *Bacillus* strains against post-harvest pathogens have demonstrated the successfully suppression of the proliferation of Fusarium dry rot in potato [19], *B. cinerea* in tomato [20], and *Colletotrichum acutatum* in banana [21].

In the present study, we aimed to gain an insight into the biocontrol potential of the endophytic bacterial strain Bvel1, by (1) investigating the taxonomic position of the bacterial strain Bvel1, (2) examining the antifungal potential of the strain Bvel1 and its secreted metabolites against *B. cinerea* in vitro, (3) identifying the antifungal compounds present in the secreted metabolites using analytical chemistry, (4) assessing the in vivo efficacy of strain Bvel1 cell cultures to control grey mold ingress and expansion in harvested table grapes, and (5) gaining an insight into the biosynthetic gene clusters involved in the synthesis of antimicrobial secondary metabolites.

## 2. Results

### 2.1. Taxonomic Classification of the Bacterial Stain Bvel1

Among the 36 endophytic bacterial strains isolated from olive tree roots, the strain Bvel1 showed the highest antagonistic activity against the post-harvest fungal pathogen *B. cinerea* and, therefore, was selected for further studies.

**Figure 1:**
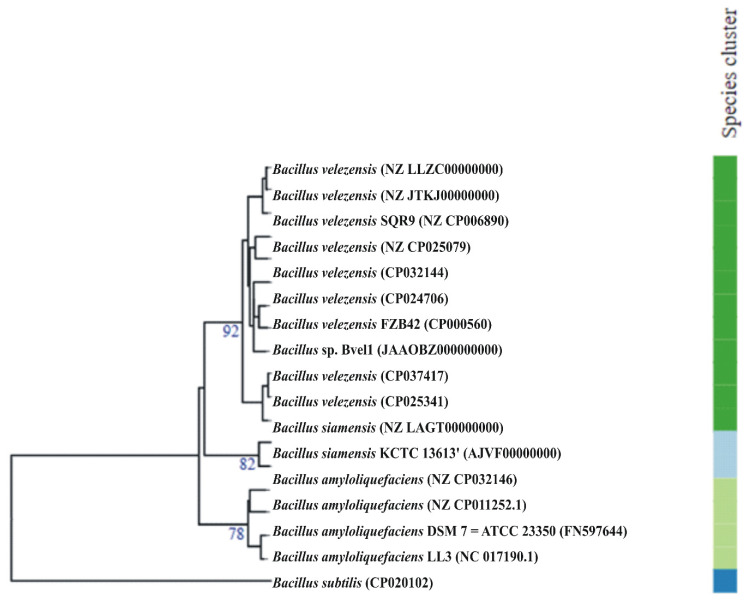
Whole-genome-based phylogenetic tree highlighting the position of *Bacillus* Bvel1 relative to other closely related *Bacillus* species. Tree inferred with FastME 2.1.6.1 [24] from genome-blast distance phylogeny (GBDP) distances calculated from genome sequences. The branch lengths are scaled in terms of GBDP distance formula d5. The numbers above the branches are GBDP pseudo-bootstrap support values > 75% from 100 replications, with an average branch support of 84%. The tree was rooted at the midpoint [24,25]. Leaf labels with different colors indicate species and subspecies clusters.

BlastN analysis based on 16S rRNA gene sequences against the public available database of NCBI (National Center for Biotechnology Information, https://www.ncbi.nlm.nih.gov (accessed on 15 June 2021) indicated that the strain Bvel1 is closely related (>99.00% identity) to strains of *B. velezensis* and *B. amyloliquefaciens*. For accurate taxonomic assignment of the *Bacillus* strain Bvel1 we compared the strain Bvel1 at a genomic level with strains belonging to the *B. amyloliquefaciens* group [22] and the type strains of the closely related *Bacillus* species deposited in the Type Strain Genome Server (TYGS) bioinformatics platform (https://tygs.dsmz.de (accessed on 13 August 2021)) [23]. As shown in Figure 1, Bvel1 was grouped into *B. velezensis* species.

Average nucleotide identity (ANI) and digital DNA–DNA hybridization (dDDH) analyses showed that the strain Bvel1 is phylogenetically related to members of *B. velezensis* species (Table 1). The similarity of 97.75% of dDDH and 79.50% of ANI values between strain Bvel1 and *B. velezensis* NRRL B-41580 confirmed that Bvel1 was affiliated to *B. velezensis* species (Table 1). The highest ANI and dDDH values were observed for *B. velezensis* LB002.

The whole genome sequences of strains Bvel1 and LB002 were compared using progressive MAUVE (Figure S1, Supplementary Materials). The currently available plasmid Bvel1 sequence exhibited a high identity with an unnamed plasmid of *Bacillus velezensis* 10075 (Figure S1, Supplementary Materials). Metabolic features of the strain Bvel1 were investigated using KEGG, COG, and CAZy. A significant proportion of the proteins could be assigned to the different categories of KEGG and COG supporting housekeeping and non-housekeeping functions (Figures S2 and S3, Supplementary Materials). It is worth noting that a significant percentage of the predicted proteins were of unknown function.

### 2.2. In Vitro Antagonistic Activity of Bacillus velezensis Bvel1 and Its Secreted Metabolites against B. cinerea

The disease suppressive potential of Bvel1 was assessed as its growth inhibitory activity against a *B. cinerea* strain, using a dual-culture assay. In all repetitions of the experiment, a clear zone of >0.7 cm was formed between the fungus and the *Bacillus* inoculum suppressing fungal mycelia radial growth by 70% compared to the control (Figure 1A). The width of the clear zone remained unaffected for at least 5 days.

**Figure 2:**
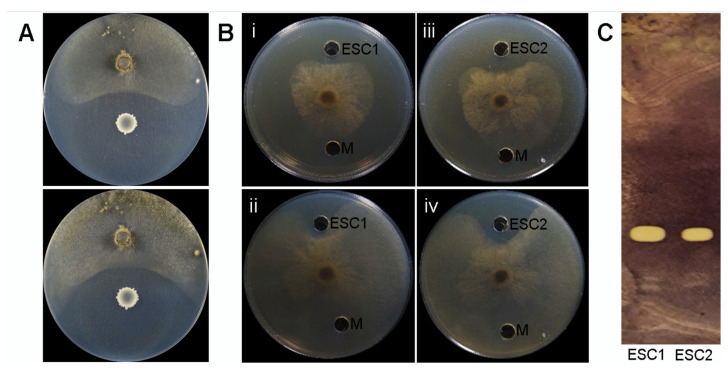
Antifungal activity of Bvel1 culture and its extracted secreted compounds (ESC) against *B. cinerea*. ESC1 and ESC2 represent extracted secreted compounds of Bvel1 when grown singly and paired with *B. cinerea*, respectively. (**A**) Direct antifungal activity of *Bacillus velezensis* Bvel1 using a dual culture assay at 3d (**top**) and 5d (**bottom)** of the interaction. (**B**) Antifungal activity of ESC1, ESC2, and M (methanol) using a well-diffusion confrontation assay at 3 days (i, iii) and 5 days of interaction (ii, iv). (**C**) TLC- bioautography using ESC1 and ESC2.

The agar diffusible compounds secreted by Bvel1 when grown individually or during interactions with *B. cinerea*, referred to as ESC1 and ESC2, respectively, were also evaluated for their antifungal activity using a well-diffusion confrontation assay and a TLC- bioautography method. The results suggested that the antifungal metabolites present in both ESCs were diffused into the agar and retained their antifungal activity for at least 5 days (suppressing expansion and growth of the fungal colony) (Figure 2B). The antifungal metabolites present in both ESCs were further characterized by TLC–bioautography using *B. cinerea* as an indicator (Figure 2C). Data revealed that both ECSs formed a strong inhibition zone suppressing conidial germination with an identical Rf value (0.55) (Figure 2C), suggesting that similar antifungal metabolites are produced and secreted by the strain Bvel1, either constitutively or during confrontation with *B. cinerea*.

### 2.3. UHPLC-HRMS Analysis of Secreted Metabolites Produced by Strain Bvel1

Ultra-high-performance liquid chromatography coupled to Q Exactive Orbitrap high-resolution mass spectrometry (UHPLC–HRMS) was employed for the identification of the secondary metabolites produced by the strain Bvel1. UHPLC–HRMS chemical analysis of ESC1 revealed the presence of lipopeptides such as iturin and surfactin (Table 2).

**Figure 3:**
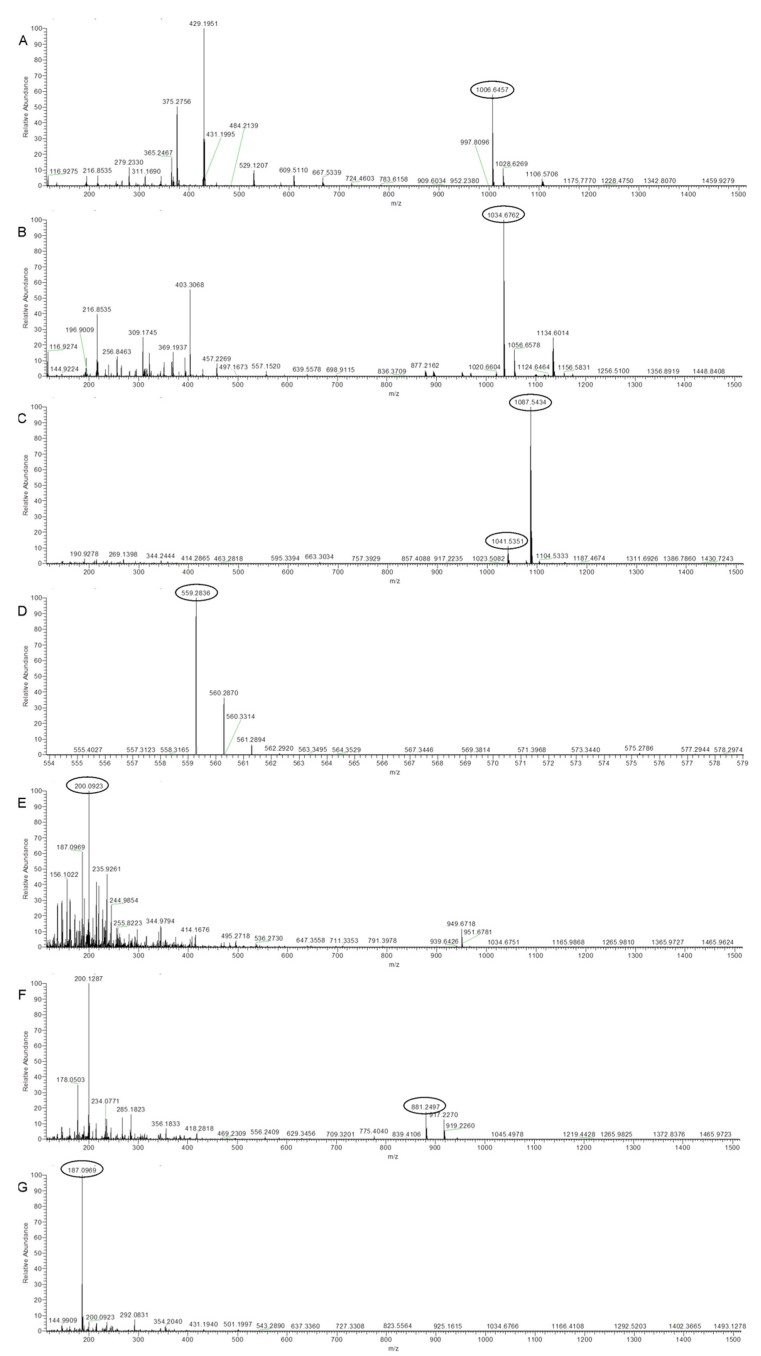
Spectra from Orbitrap high-resolution mass spectrometry (UHPLC-HRMS) analysis of the compounds secreted by Bvel1. (**A**) Surfactin A -C13, (**B**) Surfactin A -C15, (**C**) Iturin A2 -C14, (**D**) Oxydifficidin, (**E**) L-dihydroanticapsin, (**F**) Bacillibactin, and (**G**) Azelaic acid. The *m*/*z* of the compounds mentioned are circled on the spectra.

The annotation of the compounds was based on the accurate mass (±5 ppm), as well as the isotope distribution. The accurate masses obtained from ions *m*/*z* 1041.5351 [M − H]^−^ and *m*/*z* 1087.5434 [Μ + FA − H]^−^ (adduct with formic acid) revealed the presence of a compound with the molecular formula C_48_H_74_N_12_O_14_, indicating the presence of iturin A2 (Figure 3C). In addition, HRMS analysis revealed the presence of compounds with the elemental composition of C_51_H_89_N_7_O_13_ and C_53_H_93_N_7_O_13_, and ions with *m*/*z* 1006.6457 [M − H]^−^ and *m*/*z* 1034.6762 [M − H]^−^, indicating the presence of surfactin A-C13 and surfactin A-C15, respectively (Figure 3A,B). The compounds with *m*/*z* 881.2488 [M − H]^−^ and *m*/*z* 559.2836 [M − H]^−^ were attributed to the siderophore bacillibactin and the antibacterial polyketide oxydifficidin, respectively. The compound with *m*/*z* 200.0923 [M − H]^−^ was assigned to the antibacterial and antifungal component of L-dihydroanticapsin [33]. Ion signals at a *m*/*z* value of 187.0969 were assigned to azelaic acid (Figure 3G).

### 2.4. Preventive and Curative Action of Strain Bvel1 against B. cinerea on Grape Berries

**Figure 4:**
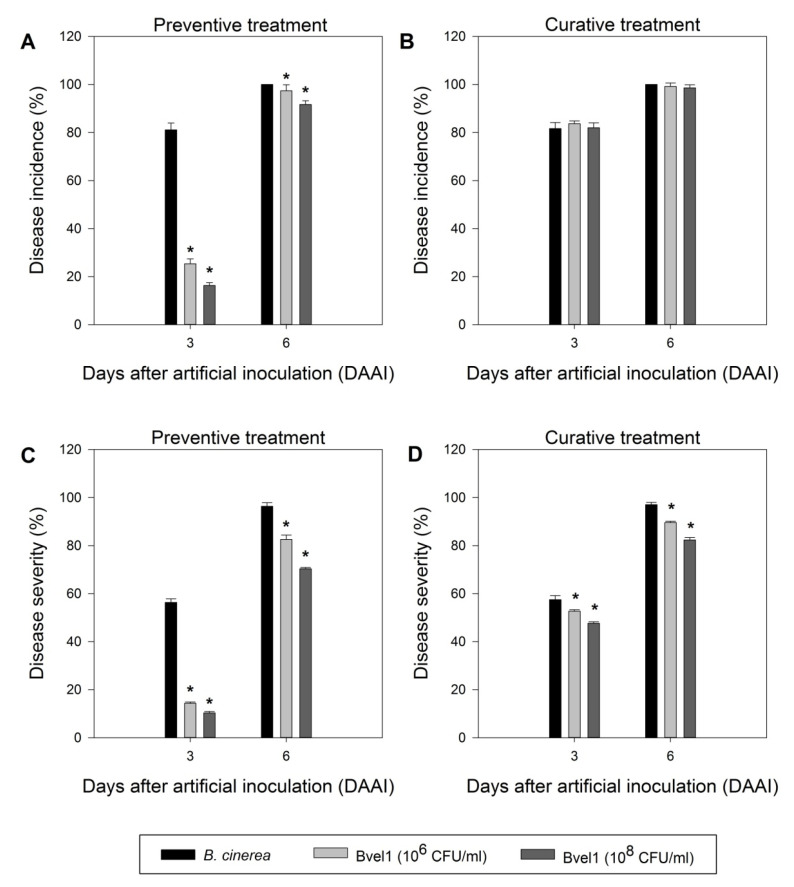
The effect of preventive and curative treatment with different concentrations (10^6^ and 10^8^ CFU/mL) of endophytic bacterial strain Bvel1 against grey mold on berries. (**A**) Disease incidence (% infected berries) of the preventive bacterial treatment, (**B**) disease incidence (%) of the curative bacterial treatment, (**C**) disease severity (% berry area with lesions) of the preventive bacterial treatment, (**D**) disease severity (%) of the curative bacterial treatment. Data values represent the mean of 3 biological replicates ± SD after *t*-test analysis. Asterisks indicate statistically significant differences of each treatment when compared to the control (*B. cinerea*) at each incubation timepoint (*p* < 0.05).

**Figure 5:**
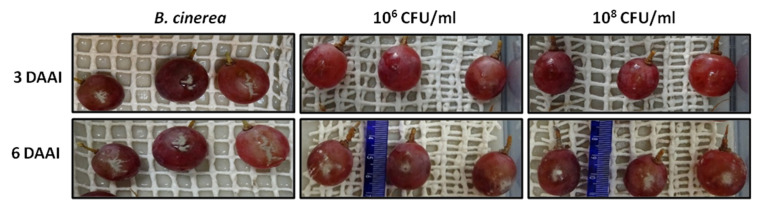
Biocontrol activity of antagonistic Bvel1 against *B. cinerea* on wounded red globe grapes. Wounded grapes treated with different concentrations (10^6^ and 10^8^ CFU/mL) of the endophytic bacterial strain Bvel1, 1 day prior to artificial inoculation with *B. cinerea*. Pictures were taken 3 days and 6 days after artificial inoculation (DAAI) and incubation at 25 °C.

To test the in planta antagonistic activity of strain Bvel1 against *B. cinerea*, we tested the inhibitory effect of a high (10^8^ CFU/mL) and medium (10^6^ CFU/mL) bacterial density culture of Bvel1 against *B. cinerea* on wounded berries either prior (preventive treatment) or after (curative treatment) artificial inoculation with a conidial suspension of *B. cinerea*. The preventive treatment showed greater effectiveness than the curative treatment, and the disease severity (% berry area covered by lesions) of the berries was reduced during the incubation period. Treatment with Bvel1 cell culture prior to *B. cinerea* inoculation strongly suppressed fungal growth and significantly reduced the incidence of grey mold (% of berries showing rot symptoms) on red globe grapes, 3 days after artificial inoculation, in a cell density dependent manner (Figure 4 and Figure 5). Disease incidence of grape berries treated with Bvel1 at different concentrations was reduced to 25.33% (1 × 10^6^ CFU/mL) and 16.33% (1 × 10^8^ CFU/mL), in comparison to 81.10% for the control (water) treatment. Furthermore, treatment with Bvel1 cells culture 24 h prior to inoculation also reduced disease severity values after 3 or 6 days of artificial inoculation, in a cell concentration dependent manner. As depicted in Figure 4, the disease severity of grey mold on the infected grape berries decreased to 14.33% and 82.57% (1 × 10^6^ CFU/mL) and to 10.33% and 70.33% (1 × 10^8^ CFU/mL) after 3 and 6 days of artificial inoculation, respectively, in comparison to 56.33% and 96.33% for the control treatment (Figure 4).

### 2.5. Bacillus velezensis Bvel1 Colonization in Wounded Berries

**Figure 6:**
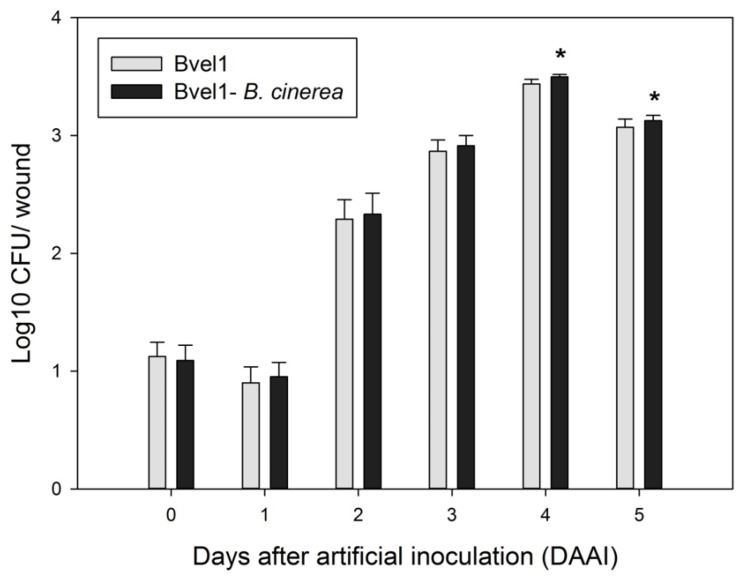
Time growth curve of Bvel1 (Log10 CFU/wound) in grape wounds (one wound in each grape) with and without the presence of the fungus *B. cinerea*. Data values represent the mean of 3 biological replicates ± SD after *t*-test analysis. Asterisks indicate the statistical difference between the two treatments (*p* < 0.05).

In the preventive treatment, the strain Bvel1 successfully colonized wounds both in the presence and absence of *B. cinerea* during the incubation period at 22 °C (Figure 6). The number of *Bacillus*-type colonies within the inoculated fruits increased rapidly from 1.09 log10 CFU/wound to 2.91 log10 CFU/wound in the presence of the fungus, and from 1.12 log10 CFU/grape to 2.87 log10 CFU/wound in the absence of the fungus, after 3 days of artificial inoculation. Bvel1 reached its maximum population after 4 days of artificial inoculation either in the presence or the absence of *B. cinerea* (3.50 log10 CFU/grape and 3.44 log10 CFU/wound, respectively), while after 5 days of artificial inoculation its population decreased slightly to 3.13 log10 CFU/wound and 3.07 log10 CFU/wound, respectively (Figure 6).

### 2.6. Genomic Insights into the Antifungal Activity of the Strain Bvel1

The genome of Bvel1 was analyzed for the presence of secondary metabolite biosynthetic gene clusters (BGCs) using antiSMASH. AntiSMASH predicted 13 putative BGCs of secondary metabolites for the strain Bvel1 genome, covering 20.4% (805.7 kb) of the whole genome (Table 3). The majority of BGCs could be assigned to known compounds, whereas five clusters probably represented novel lantipeptides, terpenes, non-ribosomal peptide synthetases (NRPS), and polyketide synthase (PKS) BGCs for which no, or low similarity, BGCs could be identified in the MIBiG database (Table 3). However, antismash-cluster blast analysis revealed that all five clusters are found in numerous strains of *B. velezensis* (data not shown). The BGCs for the sfp-dependent NRPs surfactin, fengycin, bacillaene, and bacillibactin were predicted in strain Bvel1 (Table 3). The surfactin BGC showed a similarity of 82% compared to the reference. Likewise, BGCs of bacillibactin, subtilosin A, and bacilysin were also present, with a similarity of 100% to the reference. Cluster 6 harbors two BGCs, one fengycin BGC showing 100% similarity to the reference, and one BGC showing 88%, 100%, and 100% similarities to known BGCs for the biosynthesis of iturinic-type lipopeptide iturin A, bacillomycin, and mycosubtilin, respectively. The amino acid sequence predicted by the NRPS analysis of the iturinic-type BGC indicated that it belongs to iturin A [34]. BGCs of bacilysin, macrolactin A, and difficidin were also identified, showing 100% similarity to the references.

Interestingly, genome mining also allowed the identification of genes encoding for possible antifungal CAZymes in the GH families, such as chitinase (GH18), chitosanase (GH43), endoglucanase (GH51), and β-glucosidase (GH1), which have the potential to inhibit the growth of plant pathogens [35]. The genome analysis also revealed the presence of genes (*alsD*, *alsR*, *alsS* and *bdhA*) involved in the biosynthesis of acetoin and 2,3 butanediol, volatile metabolites that act as elicitors of induced systemic resistance in plants [36].

## 3. Discussion

In recent years, there has been an increasing demand for environmentally friendly and safe methods for controlling post-harvest diseases caused by fungal pathogens. Biological control agents such as *Bacillus* sp. and their metabolites have received a lot of attention as complementary or alternative methods to the use of conventional chemical fungicides [1,3,4,5]. In this study, we showed that the endophytic *Bacillus velezensis Bvel1* can be used to control grey mold caused by *B. cinerea*.

Genome sequencing and annotation revealed that the strain Bvel1 belongs to the *B. velezensis* group. Genome mining revealed that the strain Bvel1 possesses the genetic potential to synthesize bioactive secondary metabolites such as fengycin, iturin, surfactin, bacilysin, bacillaene, difficidin, macrolactin, and bacillibactin (siderophore); the compounds endow the producing *Bacillus* with the potential to effectively antagonize bacterial and fungal plant pathogens and enhance its prospects for functioning as a biocontrol agent [34,35]. Indeed, several studies have highlighted that the successful use of numerous *Bacillus* strains as biocontrol agents is dependent, not only on their ability to colonize the target tissues, but also on whether the colonizing *Bacillus* strains possess an intact and functional biosynthetic gene cluster involved in the biosynthesis and secretion of secondary metabolites such as iturin, bacillomycin, fengycin, and surfactin [8,11,14,36,37,38,39,40,41].

Our data demonstrated that growing Bvel1 cells are capable of constitutively producing and secreting agar-diffusible compounds with a strong antifungal activity, suggesting that secondary metabolites may also be produced and secreted by Bvel1 during its colonization of grape berries. Our chemical analysis revealed that Bvel1 is capable of producing and secreting a mixture of bioactive diffusible secondary metabolites (iturin A2, surfactin A-C13 and -C15 isoforms, oxydifficidin, L-dihydroanticapsin, bacillibactin) and specialized metabolites (azelaic acid) that are known to exert a strong antifungal activity and/or trigger host plant defenses against pathogens. The iturin family lipopeptides are known for their strong antifungal activity [12] and host immune response triggering function in strawberry [42], grapevine [43], and *Arabidopsis* [10]. Among the iturin A analogues, iturin A2, in a dose-dependent manner, triggers induced systemic resistance (ISR) in chili pepper in a more efficient manner than other iturin analogues [44]. Members of the surfactin family stimulate colonization, biofilm formation, facilitate swarming motility [41], and act as activators of plant defense mechanisms against several microbial pathogens [10,42,45]. Among surfactin isoforms, the surfactin A-C15 isoform demonstrates the highest biological and elicitor activity, compared to surfactin A-C12 and -C13 isoforms [46,47]. Dihydroanticapsin, the biosynthetic precursor of bacilysin, is known for its antibacterial and antifungal activity [33]. Oxydifficidin, a derivative of difficidin, exhibits high antimicrobial activity against a wide spectrum of bacteria [12] and may trigger induced systemic resistance in plant systems [10]. The siderophore bacillibactin suppresses fungal growth by chelating the available ferric iron [12]. Azelaic acid is a known antifungal and antibacterial compound [48] that has also been described as an ISR determinant that induces resistance against the *P. syringae* pathogen in *Arabidopsis thaliana* [49]. Thus, it is evident that *Bacillus velezensis* Bvel1 is capable of producing and secreting an excellent mixture of metabolites involved in the direct suppression of plant pathogens and stimulation of plant-host defense responses against fungal and bacterial pathogens.

Our results revealed that the pre-treatment of grape berries with a culture broth containing both Bvel1 bacterial cells and their secreted metabolites resulted in the colonization of wounded grape berries and a remarkable reduction in the grey mold ingress and growth on wounds 3 or 6 days after artificial inoculation. Our data are in agreement with previous observations, where it was demonstrated that the treatment of fruits and vegetables with cell suspensions, cell cultures, CFCS, or CFCS-extracted metabolites from BCAs significantly reduced the ingress and growth of pre-harvest and post-harvest pathogenic fungi [50,51,52]. However, recent studies have demonstrated that effective biocontrol is achieved when the concentration of applied/produced pure elicitors such as plipastatin, iturin, and bacillomycin D on the target tissue is in micromolar amounts (10–50 μM) [16,44,47,53]; a concentration that is almost equal to their MIC values [16,51].

Previous studies have reported that the treatment of plant tissues (fruits, leaves) with cell suspensions, cell cultures, and cell free culture supernatant from BCAs gave very low values (<1–2 μg/g plant tissue) [14,54,55], raising questions about how to achieve the accumulation of active metabolites near or over the threshold levels required for antibiosis and/or induction of host plant defense mechanisms [54]. Recent studies have shown that bacterial colonization may generate microcolonies on the target tissue, where secondary metabolites are accumulated in micromolar amounts in microniches [56]. In line with this perspective, it could be assumed that both the secreted metabolites contained in Bvel1 culture and those possibly produced in situ by the colonizing bacteria, may allow the generation of microniches on the target tissue, where the metabolites accumulate in micromolar quantities, sufficient to trigger the host’s induced resistance and, moreover, to inhibit the ingress and growth of invading or latent fungal pathogens.

In conclusion, the results of this study revealed that the strain Bvel1 inhibited the mycelial growth of *B. cinerea* in vitro and also reduced disease incidence and disease development in red globe grapes caused by *B. cinerea*. This was likely because of its colonization ability and its capacity to constitutively biosynthesize and secrete metabolites such as iturin A2, surfactin A-C15, and azelaic acid. These attributes may allow Bvel1 to generate a hostile environment for the incoming pathogen, through competition for nutrients or space on the plant surface, generating microniches of high antibiotic activity on the fruit surface and inducing plant-mediated responses. However, further analyses will be needed to understand the functional role of metabolites produced by *B. velezensis* Bvel1 and how they are able to modulate the defense networks of plants. In this context, emphasis should be placed on metabolites such as azelaic acid, because of its potential use as a chemical elicitor of ISR-responses.

## 4. Materials and Methods

### 4.1. Isolation of Endophytic Bacteria-Microorganisms and Culture Conditions

Secondary roots were excised from four healthy olive trees (*Olea europaea* cv Koroneiki) located at the Agricultural University of Athens Experimental station (37.59 N 23.42 E). The surface sterilization of root segments (40–50 mm length) was performed according to [57]. Selected roots were rinsed with tap water, dried with absorbent papers, and then immersed in 70% ethanol solution for 1 min, followed by immersion in a solution containing 5% commercial bleach and 0.1% Tween 20 for 3 min. The tissues were immersed again in 70% ethanol for 30 s and then were rinsed 5 times with sterilized double distilled water. After rinsing, the samples were cut into smaller fragments (4–5 mm) with a sterile razor blade and crushed with sterile mortar and pestle in sterile 10 mM MgSO_4_-solution (1 g roots in 6 mL). Serial 10-fold dilutions of the suspensions were prepared, and 100 µL aliquots were plated on Nutrient agar (NA) plates. To verify the surface sterility of the roots, 100 µL of the last rinsing water was plated on NA plates amended with cycloexamide (50 μg/mL) and incubated at 28 °C for 12–14 days. Each bacterial colony was then subcultured to obtained a pure culture. Out of the 89 endophytic bacterial strains isolated from plant roots, 36 distinct morphotypes were selected for further studies. Strains were routinely maintained on NA at 4 °C, stored long-term as a 20% glycerol stock at −80 °C, and cultured in nutrient broth (NB) or NA at 30 °C for 48 h before experimental use.

The pathogen *Botrytis cinerea* Pers BPIC2585 was obtained from the Collection of Phytopathogenic Fungi (Benaki Phytopathological Institute, Kifissia, Athens, Greece) and was stored on potato dextrose agar (PDA) at 4 °C. The pathogen was cultured on PDA at 25 °C before any experimental use, and virulence was retained by regular transfers through grape berries. A conidial suspension was obtained by flooding the fungal culture with sterile distilled water, rubbing the mycelium, and filtering through a sterile nylon gauze (mesh of 200 μm). The number of conidia was counted in a hemocytometer, and the suspension was adjusted with sterile distilled water to a final concentration of 1.0 × 10^6^ conidia/mL.

### 4.2. In Vitro Antagonistic Activity of Bacillus velezensis Bvel1 against B. cinerea

The antagonistic activity of the isolated bacteria were tested in vitro by dual culture method, as previously described [58]. Briefly, a mycelial disc (5-mm diameter) was obtained from the colony edge of 7-day-old culture and was transferred onto a NA plate. Ten µL of a bacterial culture grown in Nutrient Broth (NB) for 16–18 h was spotted at a 3 cm distance from the mycelial disc. An NA plate containing the pathogen alone was used as a control. Cultures were maintained at 25 °C for 6 days, and the antagonistic effect was evaluated by measuring the distance between the edges of the growing mycelium and the antagonistic bacterium. This region was defined as an inhibition zone. The colony radius of *B. cinerea* was measured in treatment and control. The inhibition rate of mycelium growth of strain Bvel1 against strains of *B. cinerea* was calculated using the following formula: Inhibition rate (%) = ((A − B)/A) × 100; where A represents the colony diameter of the control and B represents the colony diameter of each treatment.

### 4.3. Extraction of Bacillus velezensis Bvel1 Secreted Agar-Diffusible Compounds

NA plates were inoculated with 200 μL of pre-grown bacterial culture, containing or not a mycelial disc (5-mm diameter) from the colony edge of a 7-day-old *B. cinerea* culture. NA plates containing only a bacterial inoculation were used as a control treatment. The plates were incubated at 25 °C for 6 days. The extraction of agar diffusible compounds secreted by Bvle1, henceforth referred to as Bvel1 extracts of secreted compounds (ESC) and specifically as ESC1 when grown singly or ESC2 during the interaction with *B. cinerea,* was conducted as described in the protocol of Bertrand et al. [59] and Cawoy et al. [60]. Briefly, 1.5–2.0 cm width blocks of NA medium were excised from an area located in front of the bacterial antagonist without fungal pathogen or the inhibition zone between the bacterial and fungal colony, cut into small pieces, mixed thoroughly with ethyl acetate, and put in a water-bath sonicator (Elmasonic S30H, Elma Schmidbauer GmbH, Singen, Germany) at room temperature for 30 min. The organic phase was separated and the samples were dried in a speed vacuum evaporator (Rotavapor R-114, BÜCHI Labortechnik AG, Flawil, Switzerland), re-dissolved in 1000 µL of ultrapure methanol, filtered using a 0.22 µm filter, and saved at -80 °C until testing or chemical analysis using a UHPLC-ESI HRMS (orbitrap) high resolution mass spectrometer (Thermo Scientific, San Jose, CA, USA).

### 4.4. In Vitro Antagonistic Activity of Secreted Agar-Diffusible Compounds from Strain Bvel1 against B. cinera

The well-diffusion confrontation method was used to detect and measure the antagonistic effect of ESC1 and ESC2 on *B. cinerea* mycelium growth [61]. Briefly, two holes with a diameter of 3 mm were punched aseptically with a sterile cork borer on antidiametric points of a NA plate, at a 2-cm distance from each edge. A mycelial disc (5-mm diameter) from the colony edge of a 7-day-old *B. cinerea* culture was inoculated in between. Then, 20 μL of ESC1 or ESC2 was added to one hole and 20 μL methanol to the other (control) and the NA plate was incubated at 25 °C. After the 5-day co-incubation, the zones of inhibition were recorded. The antifungal activity was determined by observing the inhibition zone of fungal growth around the hole.

### 4.5. TLC-Bioautography of Bvel1 Secreted Compounds

Thin layer chromatography (TLC) analysis of ESC1 and ESC2 and bioautography was conducted as described previously [54]. TLC was performed using silica gel 60 F254 plates (20 × 20 cm; layer thickness, 0.20 mm; Merck) and chloroform–methanol–water (65:25:4, *v*/*v*/*v*) as mobile phase. The TLC-bioautography assay was performed using the *B. cinerea* strain as an indicator strain. After migration, TLC plates were covered with PDA (0.8% *w*/*v*) previously inoculated with the indicator strain (10^5^ spores/mL) and incubated at 25 °C for 24 h. Viable cells were visualized after spraying with MTT (3-(4,5-dimethylthiazol-2-yl)-2,5-diphenyltetrazolium bromide) at a concentration of 5 mg/mL. Clear zones indicated the presence of bioactive compounds. The Rf value was calculated using the formula: Rf value = distance travelled by the solute/distance travelled by the solvent.

### 4.6. Orbitrap High Resolution Mass Spectrometry (UHPLC-HRMS) Analysis of Bvel1 Secreted Compounds

For the investigation of the chemical profiling of the ESC1 extracts a Q-Exactive Orbitrap platform (Thermo Fisher Scientific, San Jose, CA, USA) connected to a Dionex Ultimate 3000 UHPLC system (Thermo Scientific™ Dionex™, Sunnyvale, CA, USA) was employed. A Hypersil Gold UPLC C18 (2.1 × 150 mm, 1.9 μm) reversed phased column (Thermo Fisher Scientific, San Jose, CA, USA) was used. Sample analysis was carried out in both positive (ESI+) and negative (ESI-) ion mode. Eluent A (ultrapure water with 0.1% formic acid) and B (acetonitrile) were used in a gradient mode of 30 min, as follows: 0 to 21 min: 95% A: 5% B, 21 to 24 min: 5% A: 95% B, 24 to 30 min: 95% A: 5% B. The flow rate was 0.22 mL/min and data acquisition was performed on a mass range of 115–1500 Da on profile mode. The conditions for the HRMS for both negative and positive modes were set as follows: capillary temperature, 350 °C; spray voltage, 2.7 kV; S-lense Rf level, 50 V; sheath gas flow, 40 arb. units; aux gas flow, 5 arb. units; aux. gas heater temperature, 50 °C. The resolution for full scan analysis was set at 70,000, whereas for the data dependent acquisition mode the resolution was 35,000, allowing for MS/MS fragmentation of the three most intense ions. The stepped normalized collision energy was set at 35, 60, and 100. The column temperature was kept at 40 °C, while the sample tray temperature was set at 4 °C. The resulting data was processed through Compound Discovered version 2.1 (Thermo Fisher Scientific, San Jose, CA, USA). For metabolite annotation the online mzCloud library, the public chemical database PubChem (NCBI) was used, taking into consideration the isotopic and MS/MS fragmentation pattern and applying an *m*/*z* tolerance of ±5 ppm.

### 4.7. In Vivo Antagonistic Activity of Bvel1 against B. cinerea on Grape Berries

For the in vivo antifungal activity test of the strain Bvel1, red globe grapes (*Vitis vinifera* L cv. Red Globe) were surface sterilized with ethanol for 1 min, 0.1% bleach for 1 min, and 70% ethanol for 2 min, rinsed with sterile water and wounded with a sterile 21 G needle. The grapes were at the stage of berry maturation. Then, 10 μL of ddH_2_O or 10 μL of the strain Bvel1 suspension (vegetative cells, endospores and supernatant) at 10^6^ or 10^8^ CFU/mL and 10 µL of the pathogen suspension (1.1 × 10^6^ conidia/mL) was placed on the wounds. The treatments with cell cultures were added either 24 h before or 24 h after fungal inoculation. The grape berries were stored in enclosed plastic trays to maintain a high relative humidity (approximately 80%) in a ventilated cabinet at 25 °C for 6 days. There were 10 fruits with three technical replicates per treatment, and the experiment was conducted independently three times.

The number of infected grapes was recorded after 3 and 6 days of artificial inoculation, and disease incidence (DI) was calculated using the following formula: DI (%) = number of infected grape berries/total number of berries × 100. Grape berries were assessed under a stereoscope, and each berry was given a value using a 0–5 rating scale, as proposed by Calvo and co-workers [62], where: 0 = no visible symptoms; 1 = visible lesion covering less than 25% of the fruit surface; 2 = 25 to 50%; 3 = 50 to 75%; 4 = 75 to 100%. Disease severity (%) was calculated according to the Townsend–Heuberger formula [63]:

$$\mathrm{DSI}\;(\%)=\frac{\Sigma (di)}{DZ}\;\times \;100$$

where DSI is the disease severity index, *d* is the number of fruits in the scale with different disease grades, *i* is the scale (1-to-4) values, *D* is the total number of fruits examined, and *Z* is the highest scale value.

### 4.8. Colonization of the Strain Bvel1 on Wounded Grape Berries

Grape berries samples were wounded according to the previous description. The wounds were treated with aliquots (10 μL) of cell culture of the Bvel1 at 1 × 10^8^ CFU/mL alone or challenge-inoculated after 1 d with a conidial suspension of *B. cinerea* (1 × 10^6^ spores mL^−1^), and stored at 25 °C for 5 d. The tissue samples were excised with a sterile cork borer (0.4 cm diameter and 0.4 cm deep) and ground with a mortar and pestle in 10 mL of sterile phosphate-buffered saline (PBS; KH_2_PO_4_, 0.27 g/L; KCl 0.2 g/L; NaCl 8 g/L; Na_2_HPO_4_ 1.42 g/L; pH 7.0). The number of bacteria was determined by dilution-plating at 48 h after incubation on NA plates with 50 μg/mL chloramphenicol at 30 °C and expressed as Log10 CFU/wound. There were 8 fruits with three technical replicates per treatment, and the experiment was conducted independently three times.

### 4.9. Genome Sequencing

Genomic DNA was isolated from an overnight grown culture of *Bacillus* sp. strain Bvel1 using a PureLink^®^ Genomic DNA Mini Kit (Thermo Fisher Scientific, Carlsbad, CA, USA). The genome of strain Bvel1 was sequenced by SNPsaurus (Eugene, OR, USA) using an Illumina HiSeq 2000 platform, following their standard workflow for library preparation, read trimming, and assembly. This workflow used a Nextera XT DNA Library Prep Kit for library generation, followed by sequencing that generated 2x150-bp paired-end reads, followed by trimming of adaptors with BBDuk, and then assembly with SPAdes-3.12.0 using default parameters [64]. This workflow generated a total of 1,332,238 trimmed paired reads and 399.6 Mbp of sequence (>60-fold coverage). The final de novo genome of strain Bvel1 was assembled in 8 scaffolds with size of 3,945,125 bp and a plasmid with size of 14,419 bp. The whole genome project was deposited at the DDBJ/EMBL/GeneBank under the accession number JAAOBZ000000000, Bioproject: PRJNA610721, BioSample: SAMN14309916.

The chromosomal map of *Bacillus velezensis* Bvel1 was drawn using DNAPlotter [65]. Scaffolds were ordered against the reference genome of *Bacillus velezensis* LB002. Scaffolds/chromosome alignment was performed with progressive MAUVE [66], while the alignment of plasmids was calculated and drawn using the Easyfig tool [67]. Strain identity was established by The Type (Strain) Genome server (TYGS) platform available at https://tygs.dsmz.de (accessed on 13 August 2021) [23] and by calculating the digital DNA:DNA hybridization (dDDH) using the genome-to-genome distance calculator website service (GGDC 2.1) [68] and the orthologous average nucleotide identity (OrthoANI) [69].

### 4.10. Functional Genome Analysis

The proteome of the *Bacillus velezensis* Bvel1 was analyzed against the Kyoto encyclopedia of genes and genomes (KEGG) [70], cluster of orthologous genes (COG) [71], and carbohydrate-active enzymes (CAZy) [72] databases by BlastKOALA [73], WebMGA [74], and dbCAN2 [75]. Graphical representation of the main metabolic pathways of *Bacillus* sp. Bvel1 was performed with KEGG mapper [76]. A secondary metabolite analysis was performed using the antiSMASH database [77].

### 4.11. Data Analysis

The software ImageJ (https://imagej.nih.gov/ij/ (accessed on 15 June 2021)) was used for measurements on the detached fruit. Statistical analyses and plots were carried out with IBM SPSS Statistics for Windows, version 25 (IBM Corp., Armonk, NY, USA) and Sigma Plot, version 12.0 (Systat Software, San Jose, CA, USA), respectively. Data of percentages were arcsine transformed and data on bacterial population were transformed to the logarithmic scale before performing an independent samples *t* test (*p*-value < 0.05). Plots depict average values with standard deviation as error bars, and asterisks indicate statistical differences.

## Figures and Tables

**Table 1:** ANI and dDDH values obtained from the comparison of strain Bvel1 with bacterial strains closely related to the *B. amyloliquefaciens* and *B. velezensis* groups.

Bacterial Strains	OrthoANI (%)	dDDH (%)
*Bacillus velezensis* Bvel1	100.00	100.00
*B. amyloliquefaciens* DSM 7 ^T^	93.94	55.10
*B. amyloliquefaciens* LL3	93.86	54.50
*B. amyloliquefaciens* YP6	94.03	55.10
*B. velezensis* Y2	97.70	80.00
*B. velezensis* FZB42	97.70	80.10
*B. velezensis* QST713	97.62	79.20
*B. velezensis* Lzh-a42	97.82	80.00
*B. velezensis* LB002	98.70	99.84
*B. velezensis* SQR9	97.70	79.40
*B. velezensis* NRRL B-41580 ^T^	97.75	79.50

^T^ type strain.

**Table 2:** UHPLC–HRMS analysis of the secondary metabolites secreted by Bvel1.

Antibiotic Compounds	Molecular Formula	Experimental *m*/*z*	RT (min)	Δm (ppm)	Adduct	References
Surfactin A-C13	C_51_H_89_N_7_O_13_	1006.6457	22,699	2.22	[M − H]^−^	[26]
Surfactin A-C15	C_53_H_93_N_7_O_13_	1034.6762	23,945	1.39	[M − H]^−^	[27]
Iturin A2-C14	C_48_H_74_N_12_O_14_	1041.5351	14,310	−1.22	[M − H]^−^	[28]
L-dihydroanticapsin	C_9_H_15_NO_4_	200.0923	11,395	2.83	[M − H]^−^	[29]
Oxydifficidin	C_31_H_45_O_7_P	559.2836	16,950	3.01	[M − H]^−^	[30]
Bacillibactin	C_39_H_42_N_6_O_18_	881.2497	11,880	2.85	[M − H]^−^	[31]
Azelaic acid	C_9_H_16_O_4_	187.0969	11,012	2.22	[M − H]^−^	[32]

**Table 3:** Biosynthetic genes clusters (BGCs) associated with secondary metabolites found in *Bacillus velezensis* Bvel1, and their identity with known gene clusters.

Cluster Number	Synthetase	Most Similar Known Cluster	MIBiG ID (% of Genes Show Similarity)	Predicted Size (bp)
1	NPRS, TransATPKS	Rhizocticin	BGC0000926_c1 (22%)	77.624
2	NRPS	Surfactin	BGC0000433_c1 (82%)	65.407
3	PKS-like	Butirosin	BGC0000693_c1 (7%)	41.224
4	Terpene	-	-	20.740
5	Lanthipeptide-class-II	-	-	28.889
6	TransATPKS	Macrolactin	BGC0000181_c1 (100%)	86.366
7	TransATPKS-NRPS	Bacillaene	BGC0001089_c1 100(%)	109.176
8	NPRS, TransATPKS-NRPS	Fengycin	BGC0001095_c1 (93%)	114.939
9	Terpene	-	-	41.246
10	T3PKS	-	-	20.739
11	TransATPKS	Difficidin	BGC0000176_c1 (100%)	106.166
12	NPRS	Bacillibactin	BGC0000309_c1 (100%)	51.789
13	NPRS	Bacilycin	BGC0001184_c1 (100%)	41.418

## Data Availability

Bacterial strain data analyzed in this study is available in the NCBI database. The *Bacillus* sp. Bvel1 whole genome project has the BioProject: PRJNA610721, BioSample: SAMN14309916, accession number JAAOBZ000000000.

## Supplementary Materials

The following are available online at https://www.mdpi.com/article/10.3390/plants10081716/s1, Figure S1: Circular map of the *Bacillus velezensis* Bvel1 genome, Figure S2: Functional analysis of the *Bacillus velezensis* Bvel1 proteome, Figure S3: Metabolic map of *Bacillus velezensis* Bvel1.
