# Supplementary material for: Genomic Analysis and Secondary Metabolites Production of the Endophytic Bacillus velezensis Bvel1: A Biocontrol Agent against Botrytis cinerea Causing Bunch Rot in Post-Harvest Table Grapes
Source: Plants (Basel). 2021 Aug 20;10(8):1716. doi: 10.3390/plants10081716 (PMC8400388; doi:10.3390/plants10081716)
Supplement: Supplementary file 1 [file plants-10-01716-s001.zip › plants-1344962-supplementary.pdf]

Article

Genomic analysis and secondary metabolites production of the endophytic *Bacillus velezensis* Bvel1: a biocontrol agent against *Botrytis cinerea* causing bunch rot in post-harvest table grapes

Kallimachos Nifakos [1,2], Polina C. Tsalgatidou [1], Eirini-Evangelia Thomloudi [1], Aggeliki Skagia [1], Dimitrios Kotopoulis [1], Eirini Baira [3], Costas Delis [2], Konstantinos Papadimitriou [4], Emilia Markellou [5], Anastasia Venieraki [6,*] and Panagiotis Katinakis [1,*]

[1] Laboratory of General and Agricultural Microbiology, Crop Science Department, Agricultural University of Athens, Iera Odos 75, 11855 Athens, Greece; k.nifakos@go.uop.gr (K.N.); polinatsal@gmail.com (P.C.T.); e.e.thomloudi@gmail.com (E.-E.T.); Angeliki.Skagia@warwick.ac.uk (A.S.); drkotopoulis@gmail.com (D.K.)
[2] Department of Agriculture, University of the Peloponnese, 24100 Kalamata, Greece;
delis@us.uop.gr
[3] Laboratory of Toxicological Control of Pesticides, Scientific Directorate of Pesticides' Control
and Phytopharmacy, Benaki Phytopathological Institute, 8 St. Delta Street, Kifissia, 14561 Athens, Greece; e.baira@bpi.gr
[4] Department of Food Science and Technology, University of the Peloponnese, 24100 Kalamata, Greece; kostas.papadimitriou@gmail.com
[5] Scientific Directorate of Phytopathology, Benaki Phytopathological Institute (BPI), 14561 Athens, Greece; e.markellou@bpi.gr
[6] Laboratory of Plant Pathology, Crop Science Department, Agricultural University of Athens, Iera Odos 75, 11855 Athens, Greece
* Correspondence: venieraki@aua.gr (A.V.); katp@aua.gr (P.K.)

Supplementary material

Figure S1

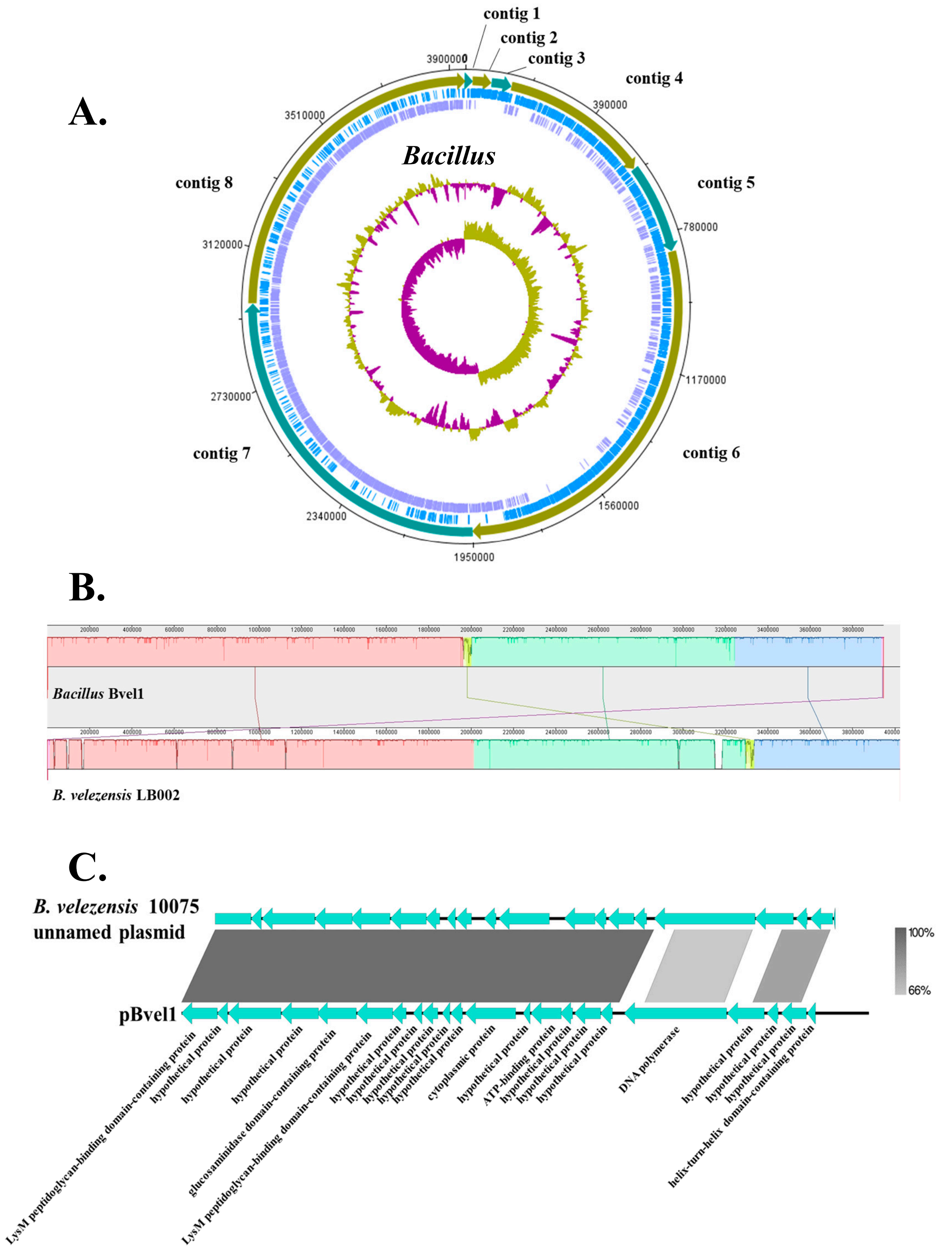

Figure S1. Circular map of the *Bacillus velezensis* Bvel1 genome **(A)**. Each ring represents specific genomic features appearing from the periphery to the center of the map: Genomic contigs in order (olive/emerald); Forward CDSs (blue); Reverse CDSs (lavender); %GC plot; GC skew. Alignment of *Bacillus* sp. Bvel1 contigs against the complete genome sequence of *B. velezensis* LB002 by progressive Mauve **(B)**. Local collinear blocks (LCBs) of conserved sequences between the two strains are represented by rectangles of the same color. Connecting

lines can be used to visualize synteny or rearrangement. The level of conservation is equivalent to the level of vertical color filling within the LCBs. Alignment of the currently available pBvel1 sequence against the unnamed plasmid of *B. velezensis* 10075 **(C)**. Gray shading corresponds to the level of % identity according to the color gradient presented at the right corner of the figure.

Figure S2

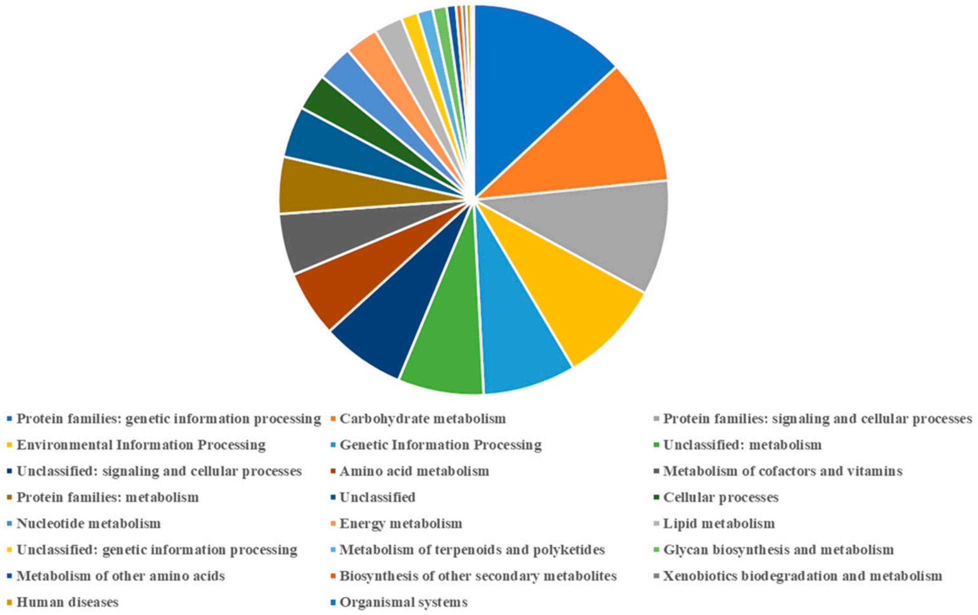

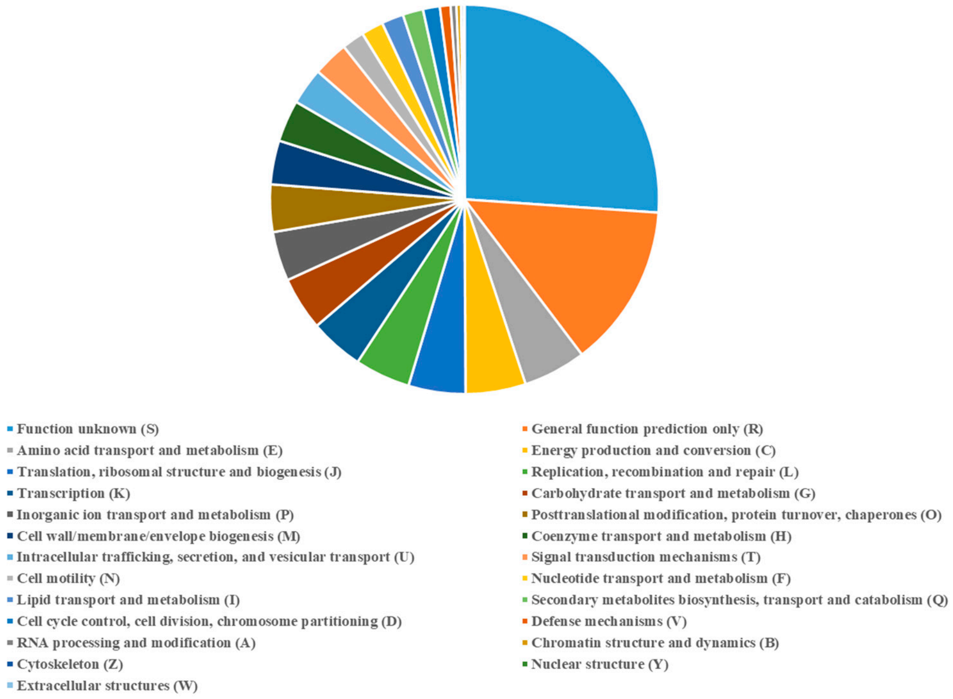

Figure S2. Functional analysis of the *Bacillus velezensis* Bvel1 proteome against the KEGG **(A)** and COG **(B)** databases.

Figure S3

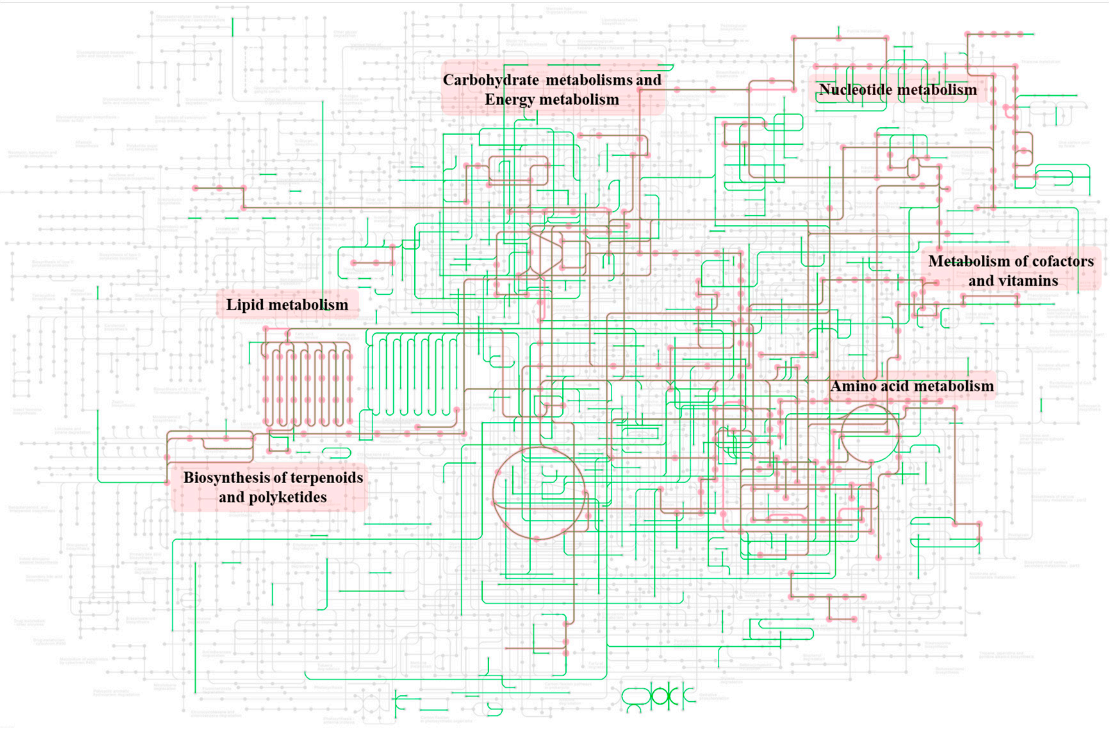

Figure S3. Metabolic map of *Bacillus velezensis* Bvel1 (green). Red lines highlight the metabolic paths indicated in the relevant text boxes.
